# An assessment of awareness of mental health conditions and its association with socio-demographic characteristics: a cross-sectional study in a rural district in Bangladesh

**DOI:** 10.1186/s12913-019-4385-6

**Published:** 2019-08-13

**Authors:** Mohammed Nazim Uddin, Sunil Bhar, Fakir M Amirul Islam

**Affiliations:** 1 Department of Statistics, Data Science and Epidemiology; Faculty of Health, Arts and Design, Swinburne University of Technology, Hawthorn, VIC 3122 Australia; 2 Department of Psychological Sciences; Faculty of Health, Arts and Design, Swinburne University of Technology, Hawthorn, VIC 3122 Australia; 3 Organisation for Rural Community Development (ORCD), Dariapur, Narail, Bangladesh

**Keywords:** Mental health conditions, Knowledge attitude and practice, Rasch analysis, Rural Bangladesh, Mental health literacy

## Abstract

**Background:**

To assess the level of awareness, knowledge and help-seeking attitudes and behaviours in relation to mental health conditions (MHCs) and associations with socio-demographic characteristics of a rural district of Bangladesh.

**Methods:**

We recruited 2425 adult samples (18–90 years) from a Cross-sectional study in Narial district of Bangladesh. Data on awareness, knowledge, help-seeking attitudes and practice in relation to six MHCs were collected. The MHCs were classified as common (depression, anxiety and drug addiction), and severe (psychosis, dementia and bipolar disorder). Associations of MHCs with socio-demographic characteristics were assessed using Chi-square tests. Rasch analysis was performed to transform the latent attribute (awareness) of MHCs from ordinal to interval scale. Multiple regression analysis was performed to determine how the socio-demographic characteristics contribute to the combined awareness score of MHCs.

**Results:**

Of 2425 participants, 17 (0.7%) were cognizant of all the awareness construct of MHCs, and 1365 (56.28%) were not aware of any of MHCs. The prevalence of awareness of MHCs such as depression (8.5%), anxiety (6.2%), psychosis (3.5%), and bipolar disorder (3.3%), was found to be very low. Awareness was significantly lower in older adults, and in women. Higher levels of education (β 1.77, 95% confidence interval (CI): 1.58–1.97) associated with common MHCs and (β 0.81, 95% CI: 0.67–0.95) those associated with severe MHCs contributed significantly to increased awareness as opposed to having no or primary level of education. Availability of sufficient funds when applied to common MHCs (β 0.43, 95% CI: 0.26–0.61) and severe MHCs (β 0.25, 95% CI: 0.13–0.38) appeared to be more effective in boosting awareness compared to unstable financial situations. Almost 100% of the participants who were aware of the MHCs demonstrated positive attitudes towards seeking medical or psychological counselling.

**Conclusions:**

Awareness of MHCs appeared to be very limited. However, knowledgeable participants were found to be very receptive to medical or psychological counselling. For improving awareness of MHCs need to conduct various intervention programs in particular those campaigns that focus on women, older adults, low SES and people up to the primary levels of education.

**Electronic supplementary material:**

The online version of this article (10.1186/s12913-019-4385-6) contains supplementary material, which is available to authorized users.

## Background

Approximately 7.3% of the global burden of disease has been attributed to mental and behavioural disorders. Most of this burden is related to unipolar depressive disorders and other mental health conditions (MHCs) including anxiety, psychosis and substance use [1]. Currently, approximately 450 million people suffer from such conditions, and it is projected that one in four individuals in the world will be affected by MHCs at some point in their lifetimes. MHCs are amongst the leading causes of ill-health and disability worldwide [2]. Globally, approximately 20% of the adults have MHCs, and low and middle-income countries have only one psychiatrist for every 1 to 4 million people [3, 4]. People with MHCs experience disproportionately higher rates of disability and mortality [5]. Individuals with major depressive disorders and schizophrenia had 40 to 60% greater chance of dying prematurely than the general population [6].

Mental health literacy (MHL), defined as “knowledge and attitudes about MHCs which aid their recognition, management and prevention” is low worldwide, but specifically low in developing countries [7]. In such societies, MHCs are believed to be consequences of familial imperfection and evil spirits [8]. Such beliefs have been purported to lead to poor utilisation and negative stigma about mental health services and treatment [9]. Unsurprisingly, poor health literacy is associated with negative disease outcomes, especially in developing countries [10–13].

The importance of health literacy on physical health is widely acknowledged in the world [14]. However, the literacy about MHCs has been neglected in both developed and developing countries [15]. The common myths in developing and developed countries are that the MHCs are not curable, caused by personal weakness, and that people with MHCs are usually violent or unstable [16]. A study from Germany reported that people were more reluctant to discuss MHCs than physical disorders with relatives and friends [17]. In the USA, many public servants did not seek treatments because they feared that MHCs would create the negative impact on their employment [18]. In developing countries, utilising services for MHCs are further blocked by stigma and beliefs about MHCs being due to sorcery or spiritual punishment, possessions by spirits and demons, genetic or family inheritance, social or moral disobediences towards ancestors or wraths of Gods [19]. A study from Nigeria showed that women in the community would be afraid to have a conversation with someone known to have mental disorders [20]. In the United Arab Emirates (UAE), women were ashamed to mention that they had a family member with mental illness, but this attitude was lower in men [21]. Moreover, a study from India reported that women thought that MHCs were family matters and should not be disclosed to other people [22]. However, in developing countries including Bangladesh and India, visiting a traditional healer for emotional problems was more common in women than in men [23]. A study revealed that in Qatar men possessed better knowledge, beliefs, and attitudes towards mental illness than women [21].

Despite the association between MHL and diseases outcomes, levels of MHL in rural regions of Bangladesh are unknown. Studies are needed to understand the level of MHL in the population and to develop targeted programs to address such levels. In the last decade, several studies have reported the prevalence of and contributing factors for depression and anxiety both in urban and rural areas in Bangladesh [24–32]. However, no study has assessed awareness, knowledge, and attitudes of seeking medical treatment regarding MHCs in Bangladesh. Rural areas in Bangladesh are characterised by traditional healing practices and an absence of mental health facilities and care. Therefore, studies on MHL are imperative to gauge and increase the level of awareness of MHCs in rural populations in Bangladesh.

The current study had two aims. First, it aimed to estimate the level of MHL in a typical rural district of Bangladesh. Second, it aimed to identify socio-demographic characteristics associated with MHL in order to identify the factors that affect rural communities and therefore inform potential interventions for improving MHL.

## Methods

### Study sample

Bangladesh is a country of 163 million people divided into 64 districts. Participants were recruited from the Narail district, which is located approximately 200 km south-west of Dhaka, the capital city of Bangladesh. The population of Narail district is 272,872, with approximately 40% of residents aged between 18 and 59 years and 19,000 (about 7%) of residents aged between 60 and 90 years. The study location was selected as it was reflective of typical rural demography in Bangladesh. The Narail district with an estimated population density of 722 people was comparable to the national rural population density of 873 people per square kilometre. Narail is not at the extremity of remote locations nor is it a catchment of a metropolis such as Dhaka [33]. The area of Narail is 381.76 km2, located in between 23°02′ and 23°17′ north latitudes and in between 89°23’and 89°37′ east longitudes. The district is surrounded by Lohagara and Salikha upazilas on the north, Kalia and Abhaynagar upazilas on the south, Lohagara upazila on the east, and Bagherpara and Jessore Sadar upazilas on the west. The ratio of male to female (48.5 to 51.5) resembled quite well with that of Bangladesh (48.9 to 51.1) [34]. 72.3% of sample data attained primary education or above as opposed to 72.9% [35] of the national population, while 27.7% of it had no education, which was comparable to the national 27.1% [35]. In the make-up of the population with respect to marital status, the Married group of Narial sample data (79.9%) was fairly proximate to the national level (80.01%) [36]. With respect to the availability of funds, a determinant for socio-economic condition, the sample population having insufficient funds some / most of the time accounted for 32.2 and 23.2% [37] in Bangladesh. In summary, the socio-demographic make-up of the Narail district delineated well the typical characteristics of a rural district of Bangladesh. Moreover, researchers carried out studies [32, 38, 39] in Narail district earlier and motived to advance further studies based on the population of Narail district.

### Sample size and statistical power

The sample comprised 2425 participants, aged between 18 to 90 years from the Narail district. The sample including 1147 older adults and 1278 adults. Prior data indicate that the prevalence of severe depression was 21% in older adults aged 60 years or above, and 6.5% in adults aged between 18 and 60 years [40]. We assumed a margin of error of 5% in prevalence rates for older adults, and of 3% for adults when estimating the true prevalence of severe depression for each cohort in this rural area [40]. Using a significance level of 0.05 and statistical power above 80%, a required sample size of 1128 was needed for the older adult cohort and 1283 for the adult group.

The sample size of 2425 was sufficiently large enough to detect a minimum 5% difference in proportion of attaining awareness or knowledge of MHCs related items between men and women; no schooling and primary or secondary level of education; have sufficient funds most of the time and insufficient funds some of the time (statistical power 90%, *p* = 0.05).

### Sampling frame

A multi-stage cluster random sampling technique was used for this study. Three unions from a total of 13 and one ward from a total of 9 wards of Narail upazilla were randomly selected at level 1. Two to three villages or mohalla from each selected union or ward were randomly selected at the level 2. About 120 older adults and 150 adults were interviewed from each of the villages. Recruitment strategy and quality assurance in data collection were described in details previously [38]. In brief, all team members participated in an intensive 2-day training programme in Narail before the commencement of the survey. The purpose of the training was to outline the rationale of the study, and the procedures and potential difficulties associated with data collection. The interviewers were instructed to visit every household within the randomly selected villages and to interview one household member of an older adult first. If none were available in this subgroup, the interviewers were approached an adult person of that household. If there was more than one male or female adult in the same household, one individual was selected, based on who was born closer to January. However, to maintain an approximately equal number of males and female participants, one female was interviewed immediately after a male participant. The recruitment started from a corner of a village and continued until the recruitment of a maximum of 250 participants was reached for a large village where the number of eligible participants were greater than 250. In case of fewer than 250 households in a village, the requirement continued to the adjacent village to reach the number to 250.

### MHCs measures

Given the relative lack of validated MHL data, the specific items measuring MHL were sourced from the National Survey of Mental Health Literacy and Stigma in Australia [41]. A questionnaire comprising these items assessed participants’ awareness of six MHCs (depression, bipolar disorder, anxiety, psychosis, dementia, and drug addiction) by asking if they had ever heard of these conditions with a possible response of “yes” or “no”. Based on their responses, they were asked to list at least one symptom of these conditions, this provided insight into the level of knowledge they possessed. Attitudes towards the use of treatment was assessed by asking the question “do they or their relatives need treatment?” with a possible response of “yes” or “no”. Participants were also asked if they or their relatives had ever experienced any MHCs, and if so, if they had undertaken treatment from a “medical doctor”, “psychologist” or others such as “spiritual persons”.

### Outcome variables

The outcome variables were determined by the level of awareness of MHCs divided into two groups, common (depression, anxiety and drug addiction) and severe (psychosis, dementia, and bipolar disorder) MHCs. Knowledge was measured based on whether the participants could identify at least one symptoms of the MHCs given that they were aware of the conditions. Attitudes were defined as positive if the participants who were aware of the MHCs were in favour of taking medical treatment or psychological counselling. Practice was defined based on whether participants’ themselves or their relatives with MHCs had undertaken medical treatment or psychological counselling.

### Socio-demographic covariates

Demographic details for age was categorised into adult (18 to 59 years) and older adult (60 to 90 years) groups. Level of education was categorised into five categories: no schooling, primary school level of education (grade 1 to 5), secondary school level of education (grade 6 to 10), school secondary certificate (SSC) or higher secondary certificate (HSC), bachelor’s degree or above. SES was assessed according to Cheng et al. [42] asking whether “over the last twelve months, concerning household food consumption, how would you classify your socioeconomic status?” The possible answers were: (i) insufficient funds for the whole year; (ii) insufficient funds some of the time; (iii) neither a deficit nor surplus (balance); and (iv) sufficient funds most of the time. Occupation was categorised as students, housewives, landowners, labourers, business men or women, government or non-government employees and retired persons.

### Statistical analyses

Participant’s sociodemographic characteristics including age, gender, level of education, occupation and SES were reported using descriptive statistics. Association of MHCs with gender, levels of education and other categorical factors were evaluated using Chi-square tests. The linear-by-linear association option in Chi-square test was used to report trends for ordinal categorical variables. Rasch Analysis is a unique approach of mathematical modelling based upon a latent trait and accomplishes stochastic (probabilistic) conjoint additivity (conjoint means measurement of persons and items on the same scale and additivity is the equal-interval property of the scale) [43]. Rasch models are used when a set of questionnaire items are intended to be summed together to provide a total score. In this study, Rasch analysis [44] was performed to compute the person measures based on the awareness for common and severe MHCs on a logarithmic scale and termed as “awareness score”. The negative value of an awareness score indicates the person has below average awareness, zero value indicates average and positive value indicates above average level of awareness of the items. Differentials of the measures of awareness and help-seeking attitudes and behaviour in relation to MHCs were evaluated across different major categories of age, gender, the level of education and other socioeconomic factors using Pearson Chi-square tests of independence. Multiple regression analysis techniques were used to evaluate the direction and strength of the effects of socio-economic factors gender, age, SES and education in predicting awareness of underlying MHCs. Data were analysed using RUMM2030 [45] and SPSS 24.0 [46].

## Results

Mean (SD, range) age of the participants was 52.0 years (17, 18–90). The demographic makeup of the total participants was 48.5% men and 51.5% women. As to the educational attainment, a considerably large proportion (66.6%) of population’s highest level of education was very modest, where 27.6% did not have any formal education and 39% completed primary education. About 14 and 16% of participants earned secondary and SSC/HSC degree respectively, while only 4% had their highest level of degree bachelor or above. The disparity between the educational distribution of adults and older adults is evident from the fact that older adults have relatively more percentage of people without education and less percentage of people with secondary education or higher as opposed to the adults. Socio-economic condition of the participants reflected that majority (about 52.9%) of them had financial stability (43% accounting for balance and 9.9% accounting for sufficient funds most of the time), while 32.2% reported occasional instability and 15.1% had precarious financial situations. Two major occupations in the overall participants were homemakers (40%) and retired persons (22.1%). Overall 5.6% were engaged in government or non-government employment, comprised of 7.6% in the adults age group and 3.5% older adults age group. Majority of the participants were married, and the second largest matrimonial class was widow with differential proportions in adults (3%) and older adults (32%). The socio-demographic characteristics of the participants by two age groups (adults and older adults) are shown in (Table 1).

**Table 1:** Sociodemographic characteristic of adults and elderly in Narail district in Bangladesh

Characteristic	Total (2425)	Adults, *n* = 1278 (52.7%)	Older adults, *n* = 1147 (47.3%)
	Mean (SD)	Mean (SD)	Mean (SD)
Age (in years)	52 (17.0)	38 (9.0)	67 (7.4)
Gender	Number (%)	Number (%)	Number (%)
Female	1249 (51.5)	675 (52.8)	574 (50.0)
Male	1176 (48.5)	603 (47.2)	573 (50.0)
Level of education (in years)			
No education	671 (27.7)	171 (13.4)	500 (43.6)
Primary (1–5)	946 (39.0)	510 (39.9)	436 (38.0)
Secondary (6–9)	327 (13.4)	238 (18.6)	89 (7.8)
SSC or HSC Pass (10–12)	385 (15.9)	295 (23.1)	90 (7.8)
Degree or equivalent (13–16)	96 (4.0)	64 (5.0)	32 (2.8)
Socio-economic condition:			
Insufficient funds most of the time	367 (15.1)	172 (13.5)	195 (17.0)
Insufficient funds some of the time	781 (32.2)	400 (31.3)	381 (33.2)
Balance	1037 (42.8)	582 (45.5)	455 (39.7)
Sufficient funds most of the time	240 (9.9)	124 (9.7)	116 (10.1)
Occupation			
Student	45 (1.9)	40 (3.1)	5 (0.4)
Housewives	970 (40.0)	629 (49.2)	341 (29.7)
Land owner	217 (8.9)	130 (10.2)	87 (7.6)
Labourers	297 (12.2)	206 (16.1)	91 (7.9)
Business	222 (9.2)	165 (12.9)	57 (5.0)
Government or non-government employee	137 (5.6)	97 (7.6)	40 (3.5)
Retired	537 (22.1)	11 (0.9)	526 (46)
Marital status			
Married	1937 (79.9)	1163 (91.0)	774 (67.5)
Widow	405 (16.7)	38 (3.0)	367 (32.0)
Unmarried/never married	78 (3.2)	73 (5.7)	5 (0.4)
Divorced/separated	5 (0.2)	4 (0.3)	1 (0.1)

Overall, perceived awareness of MHCs was low ranging from 3.3% (bipolar disorder) to 42% (drug addiction). Adults were more aware of most of the MHCs than the older adults (for example, drug addiction: 52.0% of adults vs. 30.8% of older adults, dementia disorder: 33.1% of adults vs. 20.8% of older adults), which is also evident from the significant associations of age groups and MHCs. Of participants who were aware of MHCs, more than about 90% were able to identify at least one symptom with slightly lower than 80% being cognizant of bipolar disorder symptom.

Almost all the participants were in favour of treating the MHCs. Although awareness of MHCs appeared to be associated with age groups, knowledge and attitude did not exhibit any impact of age. For instance, the proportion of positive attitude towards treating depression was 99% and did not vary significantly across the two age groups (adults and older adults Table 2).

**Table 2:** Level of awareness and knowledge of MHCs, and attitudes towards treatments for mental well-being by different MHCs and age groups in Narail district in Bangladesh

Mental Health Conditions	Awareness (heard of)		Total participants		Adults and Older Adults					
		Awareness	Knowledge^b^	Attitude towards treatment	Awareness		Knowledge^b^		Attitude towards treatment ^b^	
		*n* = 2425			Adults, n = 1278	Older Adults n = 1147	Adults	Older Adults	Adults	Older Adults
		n (%)	n (%)	n (%)	n (%)	n (%)	n (%)	n (%)	n (%)	n (%)
Common MHCs	Depression	206 (8.5)	199 (96.6)	204 (99.0)	149 (11.7)	57 (5.0)^a^	143 (96.0)	56 (98.2)	148 (99.3)	56 (98.2)
	Anxiety	151 (6.2)	141 (93.4)	149 (98.7)	121 (9.5)	30 (2.6)^a^	113 (93.4)	28 (93.3)	119 (98.3)	30 (100)
	Drug addiction	1018 (42.0)	1006 (99.0)	1004 (99)	665 (52.0)	353 (30.8)^a^	659 (99.0)	347 (98.0)	652 (98.0)	352 (100)
Severe MHCs	Psychosis	85 (3.5)	76 (89.4)	85 (100)	60 (4.7)	25 (2.2)^a^	53 (88.3)	23 (92.0)	60 (100)	25 (100)
	Dementia	662 (27.3)	659 (99.5)	661 (99.8)	423 (33.1)	239 (20.8)^a^	421 (99.5)	238 (99.6)	422 (99.8)	239 (100)
	Bipolar disorder	79 (3.3)	63 (79.7)	78 (98.7)	59 (4.6)	20 (1.7)^a^	46 (78.0)	17 (85.0)	58 (98.3)	20 (100)

^a^Significant at 0.001; ^b^ knowledge is related to participants who are aware about the disorders

**Fig. 1:**
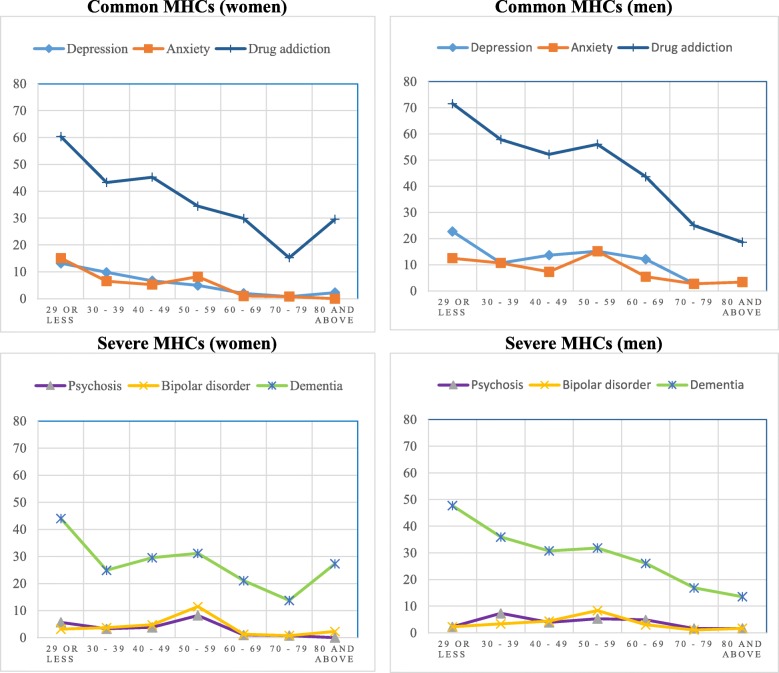
Percentage of awareness of common and severe mental health conditions in women (left) and men (right) by age group

Of the total participants 42.5% (38.0% of women vs. 47.2% of men, *p* < 0.001) were aware of any common MHCs, while 3.4% were aware of all three common MHCs. However, only 28.1% were aware of any severe MHCs while 1.3% were aware of all three severe MHCs. Among those who were aware of at least one MHCs, only 9 (0.9%) and 6 (0.9%) participants, reported that they had suffered common and severe MHCs respectively (Table 3). Awareness of any common MHCs was significantly higher in males compared to females and a similar pattern was observed in both underlying age groups for both common and severe MHCs (Fig. 1). Higher level of education (e.g.,73% of people with at least bachelor’s degree were aware of depression compared to 1% of people with no or primary level of education) and better SES appeared to be associated with higher prevalence of awareness of all the MHCs except anxiety (Table 4).

**Table 3:** Level of awareness of MHCs by gender and age groups (adults, older adults) and their associations in Narail district in Bangladesh

Mental Health Conditions	Awareness, (heard of)	Total, *n* = 2425			Adults, n = 1278			Older adults, n = 1147		
		Total	Female, *n* = 1249	Male, *n* = 1176	Total	Female, *n* = 675	Male, *n* = 603	Total	Female, *n* = 574	Male, *n* = 573
		n (%)	n (%)	n (%)	n (%)	n (%)	n (%)	n (%)	n (%)	n (%)
Common MHCs	Awareness of any MHCs	1030 (42.5)	475 (38.0)	555 (47.2)^a^	672 (52.6)	321 (47.6)	351 (58.2)^a^	358 (31.2)	154 (26.8)	204 (35.6)^a^
	Knowledge of any MHCs^b^ symptom	1020 (99)	470 (98.9)	550 (99.1)	667 (99.3)	318 (99.1)	349 (99.4)	353 (98.6)	152 (98.7)	201 (98.5)
	Awareness of all MHCs	82 (3.4)	28 (2.2)	54 (4.6)^a^	64 (5)	26 (3.9)	38 (6.3)^a^	18 (1.6)	2 (0.3)	16 (2.8)^a^
	Knowledge of all MHCs^b^ symptoms	76 (92.7)	24 (85.7)	52 (96.3)	58 (90.6)	22 (84.6)	36 (94.7)	18 (100)	2 (100)	16 (100)
	Self-reported MHCs	9 (0.9)	5 (1.1)	4 (0.7)	7 (1)	4 (1.2)	3 (0.9)	2 (0.6)	1 (0.6)	1 (0.5)
Severe MHCs	Awareness of any MHCs	682 (28.1)	334 (26.7)	348 (29.6)	438 (34.3)	217 (32.1)	221 (36.7)	244 (21.3)	117 (20.4)	127 (22.2)
	Knowledge of any MHCs^b^ symptom	676 (99.1)	331 (99.1)	345 (99.1)	433 (98.9)	215 (99.1)	218 (98.6)	243 (99.6)	116 (99.1)	127 (100)
	Awareness of all MHCs	31 (1.3)	13 (1.0)	18 (1.5)	24 (1.9)	12 (1.8)	12 (2)	7 (0.6)	1 (0.2)	6 (1.0)
	Knowledge of all MHCs^b^ symptoms	23 (74.2)	8 (61.5)	15 (83.3)	17 (70.8)	8 (66.7)	9 (75)	6 (85.7)	0 (0)	6 (100)
	Self-reported MHCs	6 (0.9)	3 (0.9)	3 (0.9)	4 (0.9)	1 (0.5)	3 (1.4)	2 (0.8)	2 (1.7)	0 (0)

^a^ Significant at 0.001; ^b^ knowledge is related to participants who are aware about the disorders

**Table 4:** Level of awareness of mental health disorders by education and socio-economic status in Narail district in Bangladesh

Mental Health Conditions	Awareness (heard of)	Education					Socio-economic condition			
		Primary (0–5), *n* = 1619	Secondary, *n* = 326	SSC or HSC, *n* = 385	Degree or equivalent, *n* = 96	*p-value for trend	In sufficient funds for at least some of the time, *n* = 1147	Balance, *n* = 1037	Sufficient funds most of the time, n = 242	*p-value for trend
		n (%)	n (%)	n (%)	n (%)		n (%)	n (%)	n (%)	
Common MHCs	Depression	17 (1.1)	25 (7.6)	94 (24.4)	70 (72.9)	< 0.001	33 (2.9)	141 (13.6)	32 (13.3)	< 0.001
	Anxiety	11 (0.7)	30 (9.2)	63 (16.4)	47 (49.1)	< 0.001	28 (2.4)	102 (9.8)	21 (8.8)	< 0.001
	Drug addiction	485 (30.2)	123 (37.6)	320 (83.3)	90 (93.8)	< 0.001	381 (33.2)	478 (46.1)	159 (66.3)	< 0.001
	Awareness of any MHCs	487 (30.1)	126 (38.5)	326 (84.7)	91 (94.8)	< 0.001	383 (33.4)	485 (46.8)	162 (67.5)	< 0.001
Severe MHCs	Psychosis	14 (0.9)	12 (3.7)	27 (7.0)	32 (33.3)	< 0.001	15 (1.3)	48 (4.6)	22 (9.2)	< 0.001
	Dementia	314 (19.4)	60 (18.3)	211 (58.4)	77 (80.2)	< 0.001	234 (20.4)	313 (30.2)	115 (47.9)	< 0.001
	Bipolar disorder	18 (1.1)	8 (2.4)	30 (7.8)	23 (24.2)	< 0.001	23 (2.0)	31 (3.0)	25 (10.4)	< 0.001
	Awareness of any MHCs	317 (19.6)	67 (20.5)	216 (56.1)	82 (85.4)	< 0.001	240 (20.9)	323 (31.1)	119 (49.6)	< 0.001
Mental Health Conditions	Awareness (heard of)	Occupation								
		Student, *n* = 45	Housewives, *n* = 978	Land owner, *n* = 217		Labourers, *n* = 282	Business, *n* = 218	Government / non-government employee, *n* = 137	Retired or unable to work, *n* = 535	**p-value
		n (%)	n (%)	n (%)		n (%)	n (%)	n (%)	n (%)	
Common MHCs	Depression	17 (37.8)^c^	51 (5.3)^a^	13 (6.0)^a^		9 (3.0)^a^	29 (13.1)^b^	60 (43.8)^d^	27 (5.0)^a^	< 0.001
	Anxiety	12 (26.7)^b^	44 (4.5)^a^	11 (5.1)^a^		10 (3.4)^a^	20 (9.0)^a^	40 (29.2)^C^	14 (2.6)^a^	< 0.001
	Drug addiction	31 (68.9)^d^	395 (40.7)^b^	114 (52.5)^c^		106 (35.7)^b^	138 (62.2)^c^	106 (77.4)^d^	128 (23.8)^a^	< 0.001
	Awareness of any MHCs	31 (68.9)^c^	398 (41.0)^b^	115 (53.0)^b^		106 (35.7)^b^	139 (62.6)^c^	111 (81.0)^d^	130 (24.2)^a^	< 0.001
Severe MHCs	Psychosis	3 (6.7)^a^	24 (2.5)^a^	4 (1.8)^a^		5 (1.7)^a^	10 (4.5)^a^	28 (20.4)^b^	11 (2.0)^a^	< 0.001
	Dementia	21 (46.7)^d^	264 (27.2)^b^	71 (32.7)^c^		65 (21.9)^b^	83 (37.4)^c^	84 (61.3)^d^	74 (13.8)^a^	< 0.001
	Bipolar disorder	4 (8.9)^b^	27 (2.8)^a^	8 (3.7)^a^		2 (0.7)^a^	8 (3.6)^a^	21 (15.3)^b^	9 (1.7)^a^	< 0.001
	Awareness of any MHCs	21 (46.7)^c^	269 (27.7)^b^	73 (33.6)^b^		66 (22.2)^a^	87 (39.2)^c^	89 (65.0)^d^	77 (14.3)^a^	< 0.001

Same symbol means there is no significant difference between the occupation and different symbol means significant difference

**indicates Fisher’s exact test was used to report *p* values for multiple pairwise comparisons

*p-value for trend indicates linear-by-linear association between awareness and underlying ordinal variables (education, socio-economic conditions)

The awareness scores obtained from Rasch analysis were categorised into two groups: persons having awareness scores greater than or equal to zero were considered to possess ‘average or above level of awareness’, and scores lower than zero were deemed to possess ‘below average level of awareness’. The mean (95% CI) awareness score of common MHCs was −4.29 (−4.38, − 4.19), and for severe MHCs was −2.18 (−2.25, − 2.12), indicating that, participants had a below average level of awareness. Only 82 (3.4%) and 113 (4.7%) participants had an awareness score above zero for common and severe awareness of MHCs respectively. Overall, this indicates they had an above average level of awareness. People who had bachelor’s or higher level of education were the sole group to have an above average awareness score for severe MHCs (multivariate adjusted mean (95% CI) score, 0.32 (0.04, 0.61)) (Additional file 1).

Multiple regression analysis was performed where *person measure* was considered as the dependent variable and gender, age, SES, education as independent variables. After controlling the effects of age, SES and level of education, awareness of common MHCs was 42% greater in males than in females (β (95% CI): 0.42 (0.25, 0.59)). Age was negatively associated with awareness implying that every increase of 10 years of age was associated with 17% lower prevalence of awareness (β (95% CI): − 0.17 (− 0.22, − 0.12)), given the other variables in the model. Having at least secondary level of education was associated with 177% higher prevalence of awareness compared to people with no education or primary level of education (β (95% CI): 1.77 (1.58–1.97)). Availability of sufficient funds most or all of the time contributed 43% higher prevalence of awareness compared to unstable financial situation (insufficient funds at least some of the time) (β (95% CI), 0.43 (0.26, 0.61)). On the other hand, gender had no significant association with the awareness of severe MHCs. Age was negatively associated with 6% lower prevalence of the awareness of severe MHCs (β (95% CI): − 0.17 (− 0.22, − 0.12)). Having at least secondary level of education was associated with 81% higher prevalence of awareness of severe MHCs compared to people with no education or primary level of education (β (95% CI): 0.81 (0.67–0.95)). Availability of sufficient funds most or all the time contributed to a 43% higher prevalence of awareness compared to unstable financial situations (insufficient funds at least some of the time) (β (95% CI), 0.25 (0.13, 0.38)) (Table 5).

**Table 5:** Influence of socio-demographic characteristics on the transformed latent trait (person measure of awareness) of MHCs

Sociodemographic characteristic	Common MHCs		Severe MHCs	
	N = 2425		N = 2425	
	β (95% CI) ^a^	β (95% CI) ^b^	β (95% CI) ^a^	β (95% CI) ^b^
Gender (men vs. women)	0.51 (0.33–0.70)^c^	0.42 (0.25–0.59)^c^	0.11 (−0.01–0.24)	0.06 (−0.06–0.18)
Age per 10-year increase	−0.33 (−0.38 - -0.28)^c^	−0.17 (−0.22 - -0.12)^c^	−0.14 (− 0.18 - -0.10)^c^	−0.06 (− 0.1 - -0.03) ^c^
SES (ref.: at least sometimes insufficient fund)	1.0 (0.82–1.18)^c^	0.43 (0.26–0.61)^c^	0.5 (0.38–0.63)^c^	0.25 (0.13–0.38) ^c^
Education (ref.: up to primary level of education)	2.16 (1.98–2.34)^c^	1.77 (1.58–1.97)^c^	0.97 (0.85–1.10)^c^	0.81 (0.67–0.95) ^c^

^a^Unadjusted β (95% CI); ^b^Adjusted β (95% CI) for variables in the model; ^c^ Significant at 0.001

## Discussion

This study was the very first kind that focused on the MHL amongst the general population in a rural district of Bangladesh. The study reported a very limited awareness about all six MHCs in general population; the lowest being 3.3% of those interviewed were aware of bipolar disorder and the highest being 42% were aware about drug addiction. More than 50% of people were not aware of any of the MHCs, whereas less than 1% people were aware of all the MHCs. Factors associated with lower awareness of MHCs were older age, female gender, lower level of education, lower level of SES, occupations of labourers or housewives. However, irrespective of age, gender, level of education or SES, most of the participants were able to identify at least one symptom of each of the MHCs items given they were aware of the MHCs, and almost all of them had positive attitudes toward medical or psychological treatments for such conditions.

The association of low level of education and female gender with low awareness of MHCs in this study appeared to align with the findings reported from in India [47]. One of the findings of this study about men being more aware of MHCs than women in both adult and older adult subgroups is quite consistent with that of the study conducted in Qatar [21]. However, another study conducted in Germany reported that the perception of awareness of MHCs among women was higher than men [17]. The disparity of awareness between Bangladeshi women and German women might be due to the fact that women in rural areas in Bangladesh are not sufficiently knowledgeable about the causes of MHCs, not well educated compared to men, do not have sufficient computer literacy, and have limited access to internet or daily newspapers. This study also revealed that education was positively associated with greater awareness of MHCs, which is quite coherent with the findings from other studies [47, 48]. Low SES [49], labourers [50] or housewives [51] were found to be associated with lower level of education, especially in the Asian countries, indicating lower level of education is one of the main barriers of attaining greater awareness of MHCs.

Another barrier in attaining awareness of MHCs can be attributed to the fact of inadequate access to medical facilities in Bangladesh. Bangladesh is the sixth most populous country in the world with approximately 50,000 medical doctors for 160 million people. Among the medical doctors, less than 1% (500) are psychiatrists, indicating less than 1 psychiatrist per 300,000 people to provide specialised treatment [52]. This ratio is much lower compared to 126 psychiatrists in Switzerland, and 44 psychiatrists in the United States [53]. For inpatient care, Bangladesh only has one dedicated mental health hospital, and 50 psychiatric units in general hospitals [52]. Such resources are insufficient to provide service for the large population in Bangladesh. The lack of visibility of such services in rural areas may contribute to the poor levels of awareness of MHCs amongst the population living in these areas.

This study provides the first reliable data on the MHL and its associated socio-demographic factors amongst general population in a typical rural district in Bangladesh. The analysis was based on a large data set collected directly through a face-to-face interview from adults and older adults. The sophisticated Rasch analysis technique was applied to quantify the MHCs item responses from a binary scale to a logarithmic scale where the binary scale suffers from identifying the real difference between two binary outcomes.

One of the criticisms of this study could be the measurement of the construct ‘socio-economic status’. The subjectivity in reporting availability of funds may affect the appropriateness and validity of socio-economic status. Secondly, the appropriateness of the KAP measure may be questioned because of the single factor/question criterion (where the answer is ‘yes’ and ‘no’) in defining the term ‘knowledge’ and ‘Attitude’. Participants were deemed knowledgeable when the participants could identify at least one symptoms of the MHCs given that they were aware of the disorders. Moreover, participants were regarded showing positive attitudes if they who were aware of the MHCs were in favour of taking medical treatment or psychological counselling. It could be argued that questions pertaining to attitudes based on ‘yes’ and ‘no’ answers may fail to capture the attitudes towards mental health adequately. Thirdly, this study did not address the potential limitation of this study is its external validity. The findings based on a single-occasion collection of data from a rural district in Bangladesh may not be truly reflective of a national perspective due to differential demographics and level of awareness of MHCs in different parts of the country.

## Conclusion

The level of awareness of MHCs was very low. All participants exhibited positive attitude towards treatments of MHCs given they were aware of the conditions. Females, lower education, older adults, and low SES groups were more likely to have a low level of awareness in most of the MHCs. The study provides evidence-based information for planning and implementation of appropriate intervention, especially in high-risk groups such as women and older adults, to increase MHL in rural Bangladesh. Public health programmes should also target those of low socioeconomic status and aim at increasing knowledge of MHCs in rural Bangladesh.

## Additional file

Additional file 1: Associations of socio-demographic characteristics with the combined awareness score of common MHCs items. (DOCX 18 kb)

## Data Availability

The datasets used and/or analysed during the current study are available from the corresponding author on reasonable request.

## Abbreviations

KAP: Knowledge, attitudes and practice
MHCs: Mental Health Conditions
MHL: Mental health literacy
