# Supplementary material for: An assessment of awareness of mental health conditions and its association with socio-demographic characteristics: a cross-sectional study in a rural district in Bangladesh
Source: BMC Health Serv Res. 2019 Aug 13;19:562. doi: 10.1186/s12913-019-4385-6 (PMC6692949; doi:10.1186/s12913-019-4385-6)
Supplement: Supplementary file 1 — Associations of socio-demographic characteristics with the combined awareness score of common MHCs items. (DOCX 18 kb) [file 12913_2019_4385_MOESM1_ESM.docx]

**Additional file 1:** Associations of socio-demographic characteristics with the combined awareness score of common MHCs items

|  | Socio- demographic characteristic | Mean (95% CI) awareness score (in logit) | |
| --- | --- | --- | --- |
|  |  | Model 1* | Model 2** |
| Gender | Female | -4.54 (-4.67, -4.41) | -4.46 (-4.57, -4.35) |
|  | Male | -4.03 (-4.16, -3.90) | -4.12 (-4.23, -4.00) |
| Age Group | Age, <30 | -3.18 (-3.46, -2.90) | -3.99 (-4.25, -3.73) |
|  | 30-40 | -3.94 (-4.15, -3.72) | -4.16 (-4.35, -3.97) |
|  | 40-50 | -4.02 (-4.24, -3.81) | -4.22 (-4.41, -4.03) |
|  | 50-60 | -3.77 (-4.09, -3.45) | -3.91 (-4.19, -3.64) |
|  | 60-70 | -4.61 (-4.78, -4.45) | -4.32 (-4.46, -4.18) |
|  | 70-80 | -5.28 (-5.53, -5.03) | -4.87 (-5.12, -4.63) |
|  | 80+ | -5.18 (-5.61, -4.74) | -4.58 (-4.98, -4.19) |
| Education | Primary including no education (0-5 ) | -5.01 (-5.1, -4.92) | -4.91 (-5.01, -4.82) |
|  | Secondary (6-9) | -4.40 (-4.61, -4.19) | -4.55 (-4.75, -4.34) |
|  | SSC or HSC Pass (10-12) | -2.29 (-2.48, -2.10) | -2.53 (-2.73, -2.33) |
|  | Degree or equivalent (13 -16) | 0.15 (-0.23, 0.53) | -0.06 (-0.45, 0.33) |
| SES | In sufficient funds for at least some of the time | -4.82 (-4.95, -4.69) | -4.40 (-4.52, -4.29) |
|  | Balance | -3.95 (-4.09, -3.81) | -4.25 (-4.36, -4.13) |
|  | Sufficient funds most of the time | -3.25 (-3.54, -2.97) | -3.97 (-4.22, -3.72) |
| Occupation | Student | -2.24 (-2.87, -1.60) | -3.99 (-4.57, -3.41) |
|  | Housewives | -4.46 (-4.59, -4.32) | -4.39 (-4.57, -4.20) |
|  | Land owner | -4.02 (-4.31, -3.73) | -3.94 (-4.23, -3.65) |
|  | Labourers | -4.70 (-4.94, -4.45) | -4.31 (-4.58, -4.05) |
|  | Business | -3.44 (-3.73, -3.16) | -4.18 (-4.47, -3.88) |
|  | Govt. or non-govt. job | -1.61 (-1.97, -1.25) | -3.38 (-3.74, -3.01) |
|  | Retired | -5.09 (-5.27, -4.91) | -4.56 (-4.76, -4.37) |

*Model 1: Unadjusted Mean (95% CI) logit,

**Model 2: Adjusted mean (95% CI) for variables in the model

Additional file (continue):

Associations of socio-demographic characteristics with the awareness score of severe MHCs items

|  | Socio- demographic characteristic | Mean (95% CI) awareness score (in logit) | |
| --- | --- | --- | --- |
|  |  | Model 1* | Model 2** |
| Gender | Female | -2.24 (-2.33, -2.16) | -2.20 (-2.29, -2.12) |
|  | Male | -2.13 (-2.22, -2.04) | -2.17 (-2.26, -2.09) |
| Age Group | Age, <30 | -1.70 (-1.89, -1.50) | -2.15 (-2.34, -1.96) |
|  | 30-40 | -2.08 (-2.23, -1.93) | -2.22 (-2.36, -2.08) |
|  | 40-50 | -2.08 (-2.23, -1.93) | -2.19 (-2.33, -2.05) |
|  | 50-60 | -1.86 (-2.07, -1.64) | -1.91 (-2.11, -1.70) |
|  | 60-70 | -2.34 (-2.45, -2.23) | -2.20 (-2.30, -2.09) |
|  | 70-80 | -2.61 (-2.78, -2.44) | -2.36 (-2.53, -2.18) |
|  | 80+ | -2.49 (-2.78, -2.19) | -2.13 (-2.42, -1.84) |
| Education | Primary including no education (0-5 ) | -2.51 (-2.58, -2.44) | -2.47 (-2.54, -2.40) |
|  | Secondary (6-9) | -2.44 (-2.59, -2.29) | -2.51 (-2.66, -2.35) |
|  | SSC or HSC Pass (10-12) | -1.26 (-1.40, -1.12) | -1.38 (-1.52, -1.23) |
|  | Degree or equivalent (13 -16) | 0.41 (0.13, 0.69) | 0.32 (0.04, 0.61) |
| SES | In sufficient funds for at least some of the time | -2.45 (-2.54, -2.36) | -2.25 (-2.33, -2.17) |
|  | Balance | -2.09 (-2.19, -2.00) | -2.24 (-2.32, -2.15) |
|  | Sufficient funds most of the time | -1.33 (-1.52, -1.13) | -1.68 (-1.87, -1.50) |
| Occupation | Student | -1.49 (-1.92, -1.05) | -2.26 (-2.68, -1.83) |
|  | Housewives | -2.23 (-2.32, -2.13) | -2.33 (-2.47, -2.19) |
|  | Land owner | -2.07 (-2.27, -1.87) | -1.85 (-2.07, -1.64) |
|  | Labourers | -2.44 (-2.61, -2.27) | -2.03 (-2.22, -1.83) |
|  | Business | -1.88 (-2.08, -1.68) | -2.07 (-2.29, -1.86) |
|  | Govt. or non-govt. job | -0.59 (-0.84, -0.34) | -1.34 (-1.6, -1.07) |
|  | Retired | -2.62 (-2.75, -2.50) | -2.42 (-2.56, -2.27) |

*Model 1: Unadjusted Mean (95% CI) logit.

**Model 2: Adjusted mean (95% CI) for variables in the model.
